# The clinical trial of alternative relugolix administration for uterine leiomyoma prior to surgically treatment: a study protocol for Non-Adverse Relugolix Administration (NARA) trial

**DOI:** 10.1186/s13063-024-07923-2

**Published:** 2024-01-19

**Authors:** Naoki Kawahara, Ryuji Kawaguchi, Konosuke Yamamoto, Kyohei Nishikawa, Motoki Matsuoka, Tomoka Maehana, Yosuke Fukui, Shoichiro Yamanaka, Sumire Sugimoto, Kana Iwai, Yuki Yamada, Hiroyuki Kurakami, Takumi Hirata, Ryuzo Takashima, Shota Suzuki, Kiyoshi Asada, Masato Kasahara, Fuminori Kimura

**Affiliations:** 1 Department of Obstetrics and Gynecology, Nara Medical University, Kashihara, 634-8522 Japan; 2 Institute for Clinical and Translational Science, Nara Medical University, Kashihara, 634-8522 Japan

**Keywords:** Relugolix, GnRH receptor antagonist, Uterine leiomyoma, NARA trial

## Abstract

**Background:**

Uterine leiomyomas are common for reproductive-aged women and affect women’s quality of life due to heavy menstrual bleeding or dysmenorrhea. Leiomyomas grow according to estradiol exposure and decrease after post-menopause. In case serious symptoms are caused by leiomyomas, pharmacotherapy or surgical treatment is proposed. Prior to surgical treatment, pharmacotherapies aimed at the reduction of leiomyoma and uterine volume or improvement of anemia are introduced to conduct minimum invasive surgery (i.e., to reduce blood loss or surgical duration). Recently, relugolix (40 mg orally once daily) as a gonadotropin-releasing hormone (GnRH) receptor antagonist has proved its sufficient efficacy in suppressing estradiol levels without the transient estradiol flare-up compared with GnRH agonist. However, long-term administration should not be permitted liable to for climacteric disorder or osteoporosis, and evidence is lacking on the actual efficacy and extent of adverse effects of the every-other-day dosing regimen. This trial aimed to prove non-inferiority in volume reduction effect on leiomyoma and safety (i.e., reduction of adverse effects) by every-other-day administration after 2 months of everyday administration compared to daily administration throughout the duration.

**Methods:**

A minimization adaptive randomized control trial (RCT) will be conducted. Patients (over 20 years old) harboring leiomyoma who will be undergoing surgical treatment will be invited to participate. Patients who are enrolled in the intervention group will receive every-other-day administration for 16 weeks after 8 weeks of daily administration. Patients who are enrolled in the control group will receive daily throughout the 24 weeks. The primary outcome is the leiomyoma volume reduction, and the secondary endpoints are the reduction of uterine volume, the occurrence of the climacteric disorder, genital bleeding days, change rate of serum hormone or bone turnover markers, and bone mineral density after 24 weeks compared to before administration.

**Discussion:**

This study aims to prove both the non-inferiority in leiomyoma volume reduction and superiority in adverse effects occurrence reduction, which will provide a novel method to escape adverse effects while maintaining the effect of leiomyoma reduction.

**Trial registration:**

Japan Registry of Clinical Trials jRCTs051230078. Registered on 26 July 2023.

## Introduction

### Background and rationale {6a}

Uterine leiomyomas are commonly detected with a prevalence reaching up to 60% of reproductive-aged women [1, 2] and affect women’s quality of life due to heavy menstrual bleeding or dysmenorrhea [3–5]. Leiomyomas grow according to estradiol exposure and decrease after post-menopause [6, 7]. In clinical practice, pharmacotherapy or surgical treatment is proposed for serious symptoms like abdominal distension, dysuria, infertility, or severe anemia due to heavy menstrual bleeding caused by leiomyomas. In the case of pharmacotherapy, a gonadotropin-releasing hormone (GnRH) receptor agonist (i.e., leuprorelin acetate or goserelin acetate) has been used to pause the menstrual cycle [8]. However, it induces a transient increase in gonadotropins and sex hormones, resulting in a clinical flare-up or temporary worsening of symptoms, and typically takes about 4 weeks to achieve a therapeutic effect [3, 9]. GnRH receptor antagonists (i.e., relugolix, elagolix, or linzagolix) are also used as newer treatments for uterine leiomyomas [10, 11]. Relugolix is a new orally active small molecule GnRH receptor antagonist [12–14] which proved its sufficient efficacy to suppress estradiol without the transient estradiol flare-up compared with GnRH agonist [15, 16]. However long-term administration should not be permitted liable to for climacteric disorder or osteoporosis. In the case of surgical treatment, pharmacotherapies like GnRH agonists or antagonists aimed at the reduction of leiomyoma and uterine volume or improvement of anemia are also introduced before the surgical treatment to conduct less invasive surgery (i.e., to reduce blood loss or surgical duration). However, the clinician often encounters cases in which menopausal symptoms are thought to be drug-related at about 8 weeks after the beginning of treatment, making it difficult to continue treatment [17]. Since the phase III trial was designed for relugolix administration to patients with leiomyoma, the leiomyoma volume as a secondary endpoint was reduced by 43.28 ± 33.85% by 12 weeks and 49.75 ± 34.33% by 24 weeks of administration compared with the baseline; we have hypothesized that everyday administration for 8 weeks and subsequently every-other-day administration of relugolix only for maintaining the effect could be a compatible method [18].

### Objectives {7}

This trial aims to prove the non-inferiority of leiomyoma reduction effect and safety evaluation (i.e., reduction of side effects) by every-other-day administration for 16 weeks after 8 weeks of everyday administration compared to throughout the duration.

### Trial design {8}

This is a phase IV study designed as a randomized, parallel-group comparative trial, with a 1:1 allocation for non-inferiority assessment.

## Methods: participants, interventions, and outcomes

### Study setting {9}

This will be a minimization adaptive randomized control trial, at the Nara Medical University Hospital in Nara, Japan. The study was conducted in accordance with the principles outlined in the Declaration of Helsinki, the International Council for Harmonization Guideline for Good Clinical Practice, and all applicable local regulatory requirements. The study, including the protocol and informed consent form, was approved by the Nara Medical University Certified Review Board (Approval Number: CRB5200002). Registration is from September 25, 2023, to August 31, 2024. The observation period will last until February 28, 2025, and the study is scheduled to end on August 31, 2025. Each patient will be provided informed consent before undergoing any study-related procedures. In the case of the study protocol modification, the information will be noticed and confirmed after approval by the review board and the Japan Registry of Clinical Trials (jRCT) modification.

### Eligibility criteria {10}

#### Inclusion criteria

**Fig. 1:**
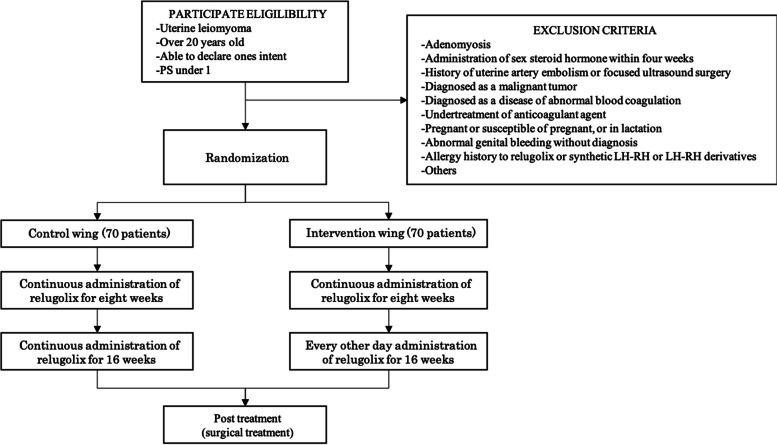
Patient disposition chart showing study participation. Of randomized patients, 70 patients will be included in the control group as the continuous administration, and the other 70 patients in the intervention group as the other day administration after 8 weeks every day of relugolix. PS, performance status

These are the requirements for participating in the study: the participant must have been diagnosed with uterine myoma, be scheduled to undergo an abdominal or laparoscopic hysterectomy or myomectomy, be a pre-menopausal female between 20 and 51 years old, can provide written consent of her own free will, and have an ECOG-PS1 rating or lower. ECOG-PS1 is a condition that limits strenuous activity but allows walking, light or sedentary work. However, it does limit strenuous activity (Fig. 1).

#### Exclusion criteria

Patients with the following factors will be excluded: adenomyosis, administration of sex steroid hormone within 4 weeks with informed consent, history of uterine artery embolism or focused ultrasound surgery, diagnosed as a malignant tumor, diagnosed as a disease of abnormal blood coagulation, undertreatment of an anticoagulant agent, pregnant or susceptible to pregnancy or in lactation; abnormal genital bleeding without a diagnosis, allergy history to relugolix or synthetic luteinizing hormone-releasing hormone (LH-RH) or LH-RH derivatives, patients who are continuously taking erythromycin or rifampicin, or others which principal investigator or collaborator decided as not appropriate (Fig. 1).

### Who will take informed consent? {26a}

The principal investigator/participating physician will give the patient an informed consent form including an explanatory document describing the research and explain it in accordance with the form. After explaining the research, giving the patient sufficient time to think about it, and confirming that the patient fully understands the content of the research, the patient will be asked to participate in the study. If the patient agrees to participate in the study, the consent document will be signed by the patient herself. One copy of the consent document will be kept by the site, and one copy will be given to the patient.

### Additional consent provisions for collection and use of participant data and biological specimens {26b}

Depending on the nature of the research, it will be conducted after review and approval by the relevant Ethics Review Committee and permission by the administrator of the implementing medical institution and after a separate explanation to the patients.

### Interventions

#### Explanation for the choice of comparators {6b}

The control group is taking 40 mg of relugolix every day for 24 weeks.

#### Intervention description {11a}

The initial dose of relugolix is 40 mg orally once daily before meals, starting on days 1–5 of the menstrual cycle until day 56. On day 57, the control group will continue taking 40 mg of relugolix every day, and the intervention group will switch to 40 mg of relugolix every other day. However, change to another group will not be permitted.

#### Criteria for discontinuing or modifying allocated interventions {11b}

The criteria for discontinuing are as follows: (1) If the subject declines to participate in the study or withdraws consent; (2) If the subject is found to be ineligible after enrollment; (3) If the primary illness is completely cured by emergency surgery or other means and there is no longer a need for continued administration; (4) If the subject has persistent heavy genital bleeding due to deterioration of the primary illness and anemia of grade 3 or higher as defined in CTCAE v5.0 Grade; (5) The following serious adverse reactions associated with study drug administration are observed. Depression: when the patient does not respond to psychiatric treatment and the main cause is considered to be the study drug. Hepatic dysfunction: AST/ALT levels of grade 2 or higher in the CTCAE v5.0 Grade definition. Angina pectoris: diagnosed during the period of study drug administration; (6) Exacerbation of side effects associated with study drug administration that makes continuation of study drug administration inadvisable; (7) If the subject develops hypersensitivity to the study drug itself; (8) If the subject becomes pregnant; (9) If the subject’s medication adherence is extremely poor or over (< 70% of the total scheduled doses or > 120% of the total scheduled doses); (10) The subject fails to come to the hospital due to relocation or other reasons and is unable to receive relugolix for more than 8 weeks; (11) The entire study is terminated; (12) The physician deems it appropriate to terminate the subject’s continued participation in the study for other reasons; and (13) If the subject has any of the following serious adverse reactions to the study drug.

#### Strategies to improve adherence to interventions {11c}

To improve adherence to medication by calculating the number of remaining doses based on the medication records in the patient logbook.

#### Relevant concomitant care permitted or prohibited during the trial {11d}

Since relugolix is a substrate of p-glycoprotein, concomitant use of erythromycin, which inhibits p-glycoprotein, or rifampicin, which induces p-glycoprotein, increases or decreases the blood concentration of relugolix, so these drugs will be restricted to concomitant use during the study period. Patients will be carefully monitored for adverse reactions to the study drug by interview and examination, and the study drug will be continued under supportive care. Supportive care includes antidepressants for depression, hepatotropic agents (ursodeoxycholic acid or stronger neo-minophagen C) for abnormal liver function, oral hemostatic agents (carbazochrome sodium sulfonate hydrate and tranexamic acid) for irregular vaginal bleeding, and iron intravenous and oral administration of iron for iron deficiency anemia associated with illicit genital bleeding are acceptable.

#### Provisions for post-trial care {30}

The post-trial care is planned to conduct myomectomy (laparotomy, laparoscopically, or hysteroscopically) or total hysterectomy (laparotomy or laparoscopically). The post-treatment is performed within 1 week after completion of 24 weeks of study drug administration. In the unlikely event that the time between the end of the study and this post-treatment is extended, patients will receive relugolix continuously during the period.

### Outcomes {12}

The primary endpoint will be the reduction of leiomyoma volume before and after the administration of relugolix. The secondary endpoints will be the reduction (%) of uterine volume compared with pre-treatment, change (score) of total Kupperman Index score at 24 weeks compared with pre-treatment, change rate (%) of bone mineral density (BMD) at 24 weeks compared with the baseline, and change rate (%) of bone metabolism markers(serum BAP) at 24 weeks compared with the baseline. The investigative assessments will be the total number of days of genital bleeding and the change in gonadotropic and sex hormone levels such as luteinizing hormone (LH), follicle-stimulating hormone (FSH), estradiol (E2), and progesterone (P4) at 24 weeks compared with the baseline. The MRI scans will be outsourced to another hospital, and tumor and uterine volume will be evaluated by the radiologist in the hospital with blind.

#### Participant timeline {13}

Eligible participants who are referred to our hospital will be recruited. Participants will be asked to complete an informed consent form by an on-site researcher if they choose to participate in the study (Fig. 1). They will complete the baseline assessments and will be randomly assigned to the intervention or control group. All patients will have follow-up visits monthly, and all outcome data will be collected by research assistants (Table 1).

**Table 1:**
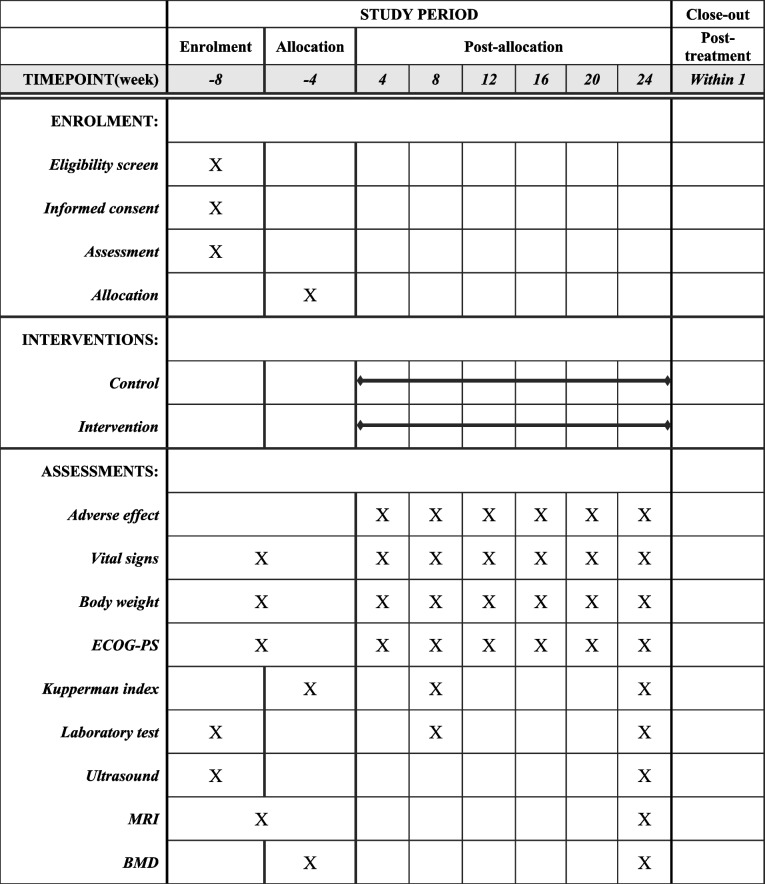
Overview of the study period (SPIRIT Fig. 2013). PS performance status, MRI magnetic resonance imaging, and BMD bone mineral density

Participants will be checked for adverse effect existence, vital sign calculation, physical measurements, and performance status evaluation every visit. Questionnaires, laboratory tests, and imaging (ultrasound, MRI, and BMD) will be conducted before, 8 weeks, and after the start of the relugolix administration

ECOG-*PS* Eastern Cooperative Oncology Group Performance Status, *MRI* magnetic resonance imaging, *BMD* bone mineral density

#### Sample size {14}

Considering the results of leiomyoma volume reduction rate after the 12 weeks of continuous administration of 20 mg and 40 mg relugolix [19], we assume a change from baseline as − 41% and − 36% for the daily and every other day groups, respectively, and a common standard deviation (SD) as 30%. Set the non-inferiority margin as 20%, the significance level as 2.5% (one-sided), and the power as 0.8; the sample size requires a minimum of 64 patients in each group. Considering a dropout rate of approximately 10%, the total number of patients in this trial will be set at 70 patients in each group, for a total of 140 patients.

#### Recruitment {15}

Post the information on the website and disseminate it to related facilities.

### Assignment of interventions: allocation

#### Sequence generation {16a}

A minimization adaptive randomization will be used for random assignment, using (1) the number of leiomyomas (2 or less or 3 or more), (2) maximum tumor diameter (5 cm or more or less), and (3) age (45 years or more or less) as adjustment factors so that there is no large bias and ensuring equal recruitment of intervention and control participants. Because the number of adjustment factors is large compared with the sample size, and a stratified permuted block randomization might not perform well, we select a minimization adaptive randomization. To reduce the risk of bias, attending physicians as researchers who will be part of the intervention will fill out all outcome data into the case report form (CRF), and data analysts will be blinded to group allocation. Eventually, this study will be no blinding, and group changes will not be allowed after the randomization.

#### Concealment mechanism {16b}

Trained research assistants will facilitate the collection of questionnaire data, physical measurements, vital signs and occurrence of adverse events, imaging assessment data, BMD, and blood tests. Imaging data includes an ultrasound examination which will be performed by the attending physician and MRI which will be outsourced and reported by its radiologist. A trained clinical laboratory technician experienced with blood collection will collect small blood samples from the patients and then analyze them immediately. Inspection items that cannot be analyzed at our hospital will be outsourced.

#### Implementation {16c}

Randomization will be conducted at the registration of patients’ data after informed consent completion. We use INDICE (UMIN Internet Data and Information system for Clinical and Epidemiological research) cloud for randomization, and initial settings will be made by the responsible allocator completely separated from the study. The INDICE is a system provided by the University Hospital Medical Information Network (UMIN) that is used for patient registration in clinical trials in Japan. It operates on the Internet to create a network-based medical research infrastructure by establishing a coordinating center on the network to support the execution of investigator-initiated medical research. Then, it is acceptable that once the initial settings are made by the person responsible for the allocation, the randomization is automatically made by entering the information during case registration by the principal investigator or collaborator.

### Assignment of interventions: blinding

#### Who will be blinded {17a}

This is an open-label trial.

#### Procedure for unblinding if needed {17b}

Not applicable.

### Data collection and management

#### Plans for assessment and collection of outcomes {18a}

Eligible participants will be invited to complete questionnaires, physical measurements, vital signs, imaging, laboratory, and bone mineral density testing as a baseline before randomization. The regular items of vital signs, body weight, PS, and the existence of adverse effects will be verified monthly after the beginning of the study. Before the administration, 8 weeks, and after 24 weeks of relugolix administration, laboratory tests, ultrasound examinations, and questionnaires will be conducted in addition to the monthly regular items. Additional examinations including laboratory tests or ultrasound examinations will be conducted in case the attending physician determined it necessary. Questionnaires include the number of days with abnormal genital bleeding and the Kupperman Index. Kupperman Index covers 11 menopausal symptoms including hot flushes (vasomotor), paresthesia, insomnia, nervousness, melancholia, vertigo, weakness, arthralgia or myalgia, headache, palpitations, and formication. Each symptom is rated on a scale from 0 to 3 for having no, slight, moderate, or severe complaints, respectively, with the highest possible total score being 51. Imaging will be admitted by magnetic resonance imaging (MRI) examination, by which select the leiomyoma with the longest diameter (excluding a calcified leiomyoma), measure in three directions in a metric system (vertical and horizontal lengths by coronal section and anteroposterior diameter by sagittal section), and calculate the volume (D1 × D2 × D3 × *π*/6) from the 3 diameters (D1, D2, and D3). Pelvic MRI scans just after the completion of the relugolix administration are acceptable with a timing error of ± 1 week. For abnormal genital bleeding, the total number of days of bleeding will be recorded using a patient diary.

#### Plans to promote participant retention and complete follow-up {18b}

Patients visit our hospital monthly, and each attending physician fills out the CRF. Revealed with severe adverse effects related to the administration of relugolix, the attending physician will examine and consult a specialist, and the principal investigator will decide whether to discontinue the trial or not, taking the participant’s wishes into consideration. The administrator of the institution will be notified, and the case will be reported to an accredited clinical research review committee. Compensation insurance will be purchased to compensate for any health problems that may occur to research subjects in relation to the clinical research. In preparation for liability, the principal investigator will purchase liability insurance.

#### Data management {19}

The data management will be conducted as follows: (1) After obtaining written consent, the principal investigator/participating physician checks eligibility and completes the CRF. (2) The principal investigator/participating physician checks for omissions on the CRF, enters the medical record number and subject ID on a collation sheet, and brings the CRF to the data center. At the data center, the principal investigator/participating physician reconfirms eligibility and performs the allocation process using the INDICE cloud. (3) After the allocation process is completed, the principal investigator/participating physician enters the allocated groups in the CRF and the collation sheet. (4) In case of withdrawal of consent, discontinuation, dropout, etc., the data center will be notified immediately.

#### Confidentiality {27}

The personal information in this study will be stored on unnetworked PCs in the medical office and laboratory or in a locked archive. The collation sheet will be managed by the personal information manager. Naoki Kawahara as the principal investigator will be appointed as the personal information manager.

#### Plans for collection, laboratory evaluation, and storage of biological specimens for genetic or molecular analysis in this trial/future use {33}

Once blood tests have been completed, any leftover samples will be transported to the obstetrics and gynecology laboratory at Nara Medical University for biobanking and future research. Samples will be used when only approved by the Research Ethics Board. Samples will be labeled with a study identification number prior to storage. The linkage document to collate patient and identification number will be stored in a vault. The principal investigator shall preserve documents related to the conduct of the study (e.g., notification documents from the hospital director, copies of various application forms and reports, research subject identification code lists, informed consent forms, case report forms, etc., and other documents or records necessary to ensure the reliability of the data) and destroy them in anonymized form 5 years after the research is published.

## Statistical methods

### Statistical methods for primary and secondary outcomes {20a}

The assessment of primary endpoint will be assessed as follows: we defined the null hypothesis as “The mean rate of change in the intervention group is more than 20% inferior to the mean rate of change in the control group,” and this will be tested with a one-sided *t*-test at a significance level of 2.5%. In addition, 95% confidence intervals for the mean rate of change are calculated for each group and for the difference between the groups. The assessment of the secondary endpoint will be analyzed as follows. (1) Change in uterine volume from before study drug administration at 24 weeks after study drug administration (%). The measured values before the start of treatment and at 24 weeks and the rate of change at 24 weeks will be summarized for each group using descriptive statistics. The 95% confidence interval of the difference between the groups will be calculated for the mean of the rate of change. (2) Change in the total score of the Kupperman Index from before the study drug administration at 24 weeks after the study drug administration (points). (3) Percent change in bone mineral density (BMD) from baseline at 24 weeks post-treatment (%). The measurements before and at week 24 of treatment and the percent change at week 24 of treatment will be summarized for each group by descriptive statistics. In addition, the null hypothesis “the mean rate of change for the intervention group is equal to the mean rate of change for the control group” will be tested with a two-sided 5% *t*-test at the significance level. In addition, 95% confidence intervals for the mean rate of change will be calculated for each group and for the difference between the groups. (4) Percentage change in bone metabolism markers (serum BAP) at 24 weeks after treatment compared to before treatment with the study drug (%). The measured values before the treatment and at 8 and 24 weeks of treatment, and the change (%) at 8 and 24 weeks of treatment will be summarized for each group using descriptive statistics, and a graph of the mean ± standard deviation will be prepared for each time point. The null hypothesis “the mean of the amount (rate) of change in the intervention group is equal to the mean of the amount (rate) of change in the control group” is tested with a two-tailed *t*-test at a significance level of 5%. In addition, 95% confidence intervals for the mean of the amount (rate) of change will be calculated for each group and for the difference between the groups. Data for each endpoint will be analyzed after the period for collecting the data has ended and the data have been fixed. The intermediate analysis will not be performed.

### Interim analyses {21b}

Because this study was designed to confirm the non-inferiority of a dose reduction of an already effective drug, it was highly unlikely that the primary endpoint would be significantly improved or worsened compared to controls.

### Methods for additional analyses (e.g., subgroup analyses) {20b}

There are no plans to conduct subgroup analyses. In case there are any modifications to the original statistical analysis plan, the plan itself and, if necessary, the research protocol will be updated accordingly. The summary report of the study will include an explanation of the updated sections and the reasons for the changes made to the statistical analysis plan.

### Methods in analysis to handle protocol non-adherence and any statistical methods to handle missing data {20c}

In terms of protocol compliance, the following cases are considered as part of the discontinuation criteria: poor drug adherence (less than 70% or more than 120% of the total scheduled doses), the inability of the patient to come to the hospital due to relocation or other reasons, and the inability to administration of relugolix for more than 8 weeks.

### Plans to give access to the full protocol, participant-level data, and statistical code {31c}

The principal investigator shall prepare a primary endpoint report (a document summarizing the results of data collection for the primary endpoint) and a summary report (a document summarizing the results of the relevant clinical research) and its summary within the following period, and publish them by recording them in the jRCT.

1. Primary endpoint report: to be prepared and published in principle within 1 year after the end of the period for collecting data on the primary endpoint.
2. Summary report and its summary: to be prepared and published in principle within 1 year after the end of the period for collecting data on all endpoints.

Full protocol can be provided upon request, but participant-level data and statistical code will be provided through anonymization after approval by the ethics review board.

### Oversight and monitoring

#### Composition of the coordinating center and trial steering committee {5d}

The facility administrator should receive periodic reports regarding the implementation plan, the decision on whether or not to conduct the study, opinions from the Certified Review Board (CRB), reporting adverse events and other illnesses (including serious illnesses) that may have a causal relationship with the research, periodic reporting every year and within 2 months after the end of each relevant period, primary endpoint report and summary report preparation, summary report public availability, and public disclosure of the summary report. The CRB is an organization approved by the Ministry of Health, Labor and Welfare. Its roles include evaluating and approving the research protocol, inspecting source documents, reporting adverse events and other illnesses (including serious illnesses) that may have a causal relationship with the research, submitting periodic reports annually and within 2 months after the relevant period has expired, submitting noncompliance reports, making recommendations for deviations from the research protocol, or revising or discontinuing the study.

#### Composition of the data monitoring committee, its role, and reporting structure {21a}

Monitoring will be conducted to confirm that the study is being conducted safely and in accordance with the protocol and that data is being collected accurately. The details of monitoring (method, timing, content, etc.) will be specified in the “Procedures for Monitoring” and conducted in accordance with the “Monitoring Plan.” Monitoring will be conducted by a specialist who is not directly involved in the conduct of the study at the site, in accordance with the “Procedures for Monitoring” and the “Monitoring Plan.”

#### Adverse event reporting and harms {22}

The safety endpoints in this study are as follows: (1) depression during the trial with the relugolix by which it is most likely caused, (2) hepatic dysfunction (aspartate aminotransferase [AST]/alanine aminotransferase [ALT] levels) indicated as grade 4 by Common Terminology Criteria for Adverse Events (CTCAE) v5.0 during treatment with the relugolix, (3) angina pectoris with the relugolix, and (4) severe anemia indicated as grade 4 by CTCAE v5.0 during treatment with the relugolix. Depression is a relatively common side effect, occurring in less than 1% of patients, but will be considered less common in this trial because half of the participants will be administered a half dose approved by the Ministry of Health, Labor and Welfare in Japan. In the event of the above adverse reaction, the patient will be promptly examined and treated by a medical specialist, and a decision will be made whether to discontinue the trial or not, taking the participant’s wishes into consideration. The administrator of the institution will be notified, and the case will be reported to an accredited clinical research review committee. Although the frequency of hepatic dysfunction is not revealed but has also been reported, then the same measures will be taken. This clinical research does not include information about genetic characteristics that can be passed on to subjects or their offspring.

#### Frequency and plans for auditing trial conduct {23}

Trial auditing will not be conducted in this trial. However, if monitoring reveals serious violations of relevant laws and regulations or deviations from the protocol, an audit may be conducted at the direction of the principal investigator.

#### Plans for communicating important protocol amendments to relevant parties (e.g., trial participants, ethical committees) {25}

The principal investigator shall promptly submit a revised research protocol and a set of documents, including a revised explanation document for the patient in accordance with the revised protocol, to the CRB for re-review and approval. The same procedure should be followed for revisions of the research protocol, etc. based on the opinions of the CRB.

#### Dissemination plans {31a}

The principal investigator has the responsibility to publish two reports, namely the primary endpoint report and the summary report. The primary endpoint report is a document that summarizes the results of data collection for the primary endpoint, while the summary report summarizes the results of the relevant clinical research. These reports, along with their summary, should be recorded in the jRCT.

## Discussion

There is no proceeding trial, at least to our knowledge, that investigated into size reduction effect by every other day administration of relugolix. The Osuga et al. [18] phase III trial aimed to compare the effectiveness of relugolix 40 mg/day with leuprorelin 1.88 or 3.75 mg monthly injections in treating uterine leiomyoma patients over 24 weeks. The trial found that daily administration of relugolix 40 mg/day significantly reduced tumor volume by 41% at 12 weeks. Thereafter, the tumor volume appeared to stabilize at 24 weeks with a 49% reduction compared to the baseline. This suggests that the volume reduction effect of leiomyoma through everyday administration of relugolix is likely to reach a plateau at 12 weeks. Adverse effects of climacteric disorder induced by relugolix administration in clinical settings occur around 8 weeks after the start of treatment. In the current trial, we expect that daily administration of up to 8 weeks, by which climacteric disorder symptoms are less likely to occur, followed by a change to every other day administration, would escape complications while maintaining the effect of leiomyoma volume reduction. Relugolix has a longer half-life, allowing for once-daily administration and improving patients’ compliance with long-term therapy [19, 20]. It may also be suitable for every other day administration.

The relugolix combination therapy is a once-daily treatment for uterine myomas or endometriosis. It comprises 40 mg relugolix, 1 mg estradiol, and 0–5 mg norethisterone acetate. The therapy helps maintain hormone base levels at about the early follicular phase concentration of the menstrual cycle, which reduces vasomotor symptoms and minimizes bone density loss. Several randomized third-tier trials have been conducted to test the effectiveness of the therapy [15, 21–23]. In the LIBERTY study, which focused specifically on myomas, both the relugolix combination and the delayed group showed significant improvements in menstrual volume, pain, bleeding distress, pelvic discomfort, anemia, and uterine volume when compared with the placebo group [15]. However, neither the relugolix combination nor the delayed group showed any significant reduction in maximum uterine myoma volume. The above trial may not be sufficient for reducing myoma volume before less invasive surgery. However, the current trial has the potential to advocate new methods of administering these combination therapies.

In conclusion, if this study confirms the non-inferiority of the primary endpoint and the superiority of the secondary endpoints of relugolix 40 mg/day for 8 weeks followed by every other day for 16 weeks compared to the standard treatment of 24 consecutive weeks, a novel protocol that can contribute both of the better economic prowess and the less burdensome for patients.

## Trial status

Protocol version number and date: version 1.2, August 16, 2023.

Start date of recruitment: October 1, 2023.

Estimated completion date of recruitment: August 31, 2025.

## Data Availability

The final trial dataset will be available to the study investigators and the Research Ethic Boards at our institution. The datasets analyzed during the current study and statistical code are available from the corresponding author upon reasonable request, as is the full protocol.

## Abbreviations

ALT: Alanine aminotransferase
AST: Aspartate aminotransferase
BAP: Bone-specific alkaline phosphatase
BMD: Bone mineral density
CRF: Case report form
CTCAE: Common Terminology Criteria for Adverse Events
DXA: Dual-energy X‐ray absorptiometry
ECOG: Eastern Cooperative Oncology Group
E2: Estradiol
FSH: Follicle-stimulating hormone
GnRH: Gonadotropin-releasing hormone
INDICE: UMIN Internet Data and Information system for Clinical and Epidemiological research
jRCT: Japan Registry of Clinical Trials
LH: Luteinizing hormone
LH-RH: Luteinizing hormone-releasing hormone
MRI: Magnetic resonance imaging
NARA trial: Non-Adverse Relugolix Administration trial
PS: Performance status
P4: Progesterone
RCT: Randomized control trial
SD: Standard deviation
